# Working memory training in children with neuropsychiatric disorders and mild to borderline intellectual functioning, the role of coaching; a double-blind randomized controlled trial

**DOI:** 10.1186/s12888-017-1274-6

**Published:** 2017-03-28

**Authors:** Sammy Roording-Ragetlie, Helen Klip, Jan Buitelaar, Dorine Slaats-Willemse

**Affiliations:** 1 Karakter Child and Adolescent Psychiatry, Nijmegen, The Netherlands; 2 Department of Cognitive Neuroscience, Radboud University Medical Centre, Donders Institute for Brain, Cognition and Behaviour, Nijmegen, The Netherlands; 3 Centre for Cognitive Neuroimaging, Donders Institute for Brain, Cognition and Behaviour, Radboud University Nijmegen, Nijmegen, The Netherlands; 4 Department of Psychiatry, Radboud University Medical Centre, Donders Institute for Brain, Cognition and Behaviour, Nijmegen, The Netherlands; 5 Karakter Child and Adolescent Psychiatry, Utrechtseweg 320, 6862 BC Oosterbeek, The Netherlands

**Keywords:** Mild to borderline intellectual functioning, ADHD, ASD, Working memory training, Coaching, Randomized controlled trial

## Abstract

**Background:**

Working memory training (WMT) has been shown to offer therapeutic benefits to both patients with Attention-Deficit Hyperactivity Disorder (ADHD) and patients with mild to borderline Intellectual Disabilities (MBID; 60 < IQ < 85). However, robust evidence for transfer effects and treatment benefits of WMT over placebo training are lacking. Owing to the nature of double-blind research designs in RCTs, children have received non-specific coaching not based on their actual training performance. Active coaching based on individual training results (such as in clinical practice) might enhance the efficacy of Cogmed WMT. Furthermore, clinical experience and the general treatment approach to these vulnerable children has shown that the intensity and duration of WMT is often too stressful. This study therefore investigated the efficacy of a less intensive, but more prolonged Cogmed WMT (including active personalized coaching and feedback) in reducing behavioral symptoms and improving neurocognitive functioning and academic achievements in children with MBID and neuropsychiatric disorders.

**Methods/design:**

A double-blind RCT with children (age 10.0–13.11) with neuropsychiatric disorders (ADHD and/or autism spectrum disorder (ASD)) and MBID (IQ: 60 < IQ < 85). Two groups (each *n* = 26) will receive Cogmed WMT (version R/M) at home or at school for 8 weeks, 4 days a week, at 30 min a day. One group will receive active personalized coaching and feedback based on their actual individual performance during Cogmed training**.** The other group will only receive general non-personalized coaching (i.e. no receive personalized coaching and feedback). Both groups will undergo a neurocognitive assessment (working memory, executive functioning, academic achievements) before and after training and complete several questionnaires (behavioral problems, parenting style) with a 6 months follow-up.

**Discussion:**

This study will add to the literature since the role of coaching in Cogmed WMT has not been studied before. It will also provide opportunities to investigate an alternative version of WMT in a large group of vulnerable children, for whom few evidence-based treatments are available. Ultimately, this will allow us to advise mental health care professionals and special education schools about the use of this type of intervention for children with MBID and neuropsychiatric disorders.

**Trial registration:**

Dutch Trial Register. NTR5223. Registration date 06–09-2015.

## Background

It is important to develop evidence-based treatments for children with MBID (IQ: 60 < IQ < 85) and neuropsychiatric disorders, as regular cognitive-behavioral therapies are often too complex for this group due to lower intellectual abilities and less developed adaptive skills. The group of children with MBID is of particular interest, since 33% may have a comorbid psychiatric disorder, the most common being ADHD and ASD [1].

WMT has been shown in several studies to offer therapeutic benefits to both patients with ADHD and patients with MBID. Recent reviews on the efficacy of Cogmed WMT have suggested Cogmed RM may be a potentially efficacious treatment for ADHD [2, 3]. Studies on the MBID-group report improvements in short-term memory (STM) and working memory (WM) as well as in academic achievements [4, 5]. A recent meta-analytic review revealed a significant overall pretest-posttest effect size for WMT for individuals with intellectual disabilities compared to controls [6]. On the other hand, 2 previous meta-analyses on non-pharmacological interventions suggest better evidence for the efficacy of cognitive training is required before these interventions may be labelled as effective interventions for ADHD [7, 8]. Rapport and colleagues [9] showed moderate improvements in STM performance in a meta-analysis of several cognitive training programs in children with ADHD when focusing on STM alone (such as Cogmed WMT). However distal transfer effects as well as training effects on other neurocognitive domains, academic achievements or behavior were negligible [9].

Extended research on the efficacy of WMT in a variety of patient groups is needed. This study is designed to examine the effect of WMT in a scientific rigorously way in children with MBID and neuropsychiatric disorders (i.e. ADHD, ASD). Previous studies on Cogmed WMT have underlined the importance of active, personalized coaching and feedback during training [3, 10]. A study by van Dongen and colleagues [10] included a younger ADHD patient group with normal intelligence. Both children treated with Cogmed and those treated with the placebo control version improved on the outcome measures. Due to the inclusion of a placebo-controlled version, children received non-personalized coaching (not based on their actual training performance). This is not consistent with the personalized coaching and feedback (based on actual performance) in clinical practice. Preliminary blind analysis from our recent study on the efficacy of Cogmed in neuropsychiatric disorders and borderline intellectual functioning (Roording-Ragetlie S, Klip H, Buitelaar J, Slaats-Willemse D. Working memory training in children with neuropsychiatric disorders and borderline intellectual fucntioning; a randomized controlled trial, unpublished raw data) has also shown that both groups, i.e. those children treated with Cogmed and those treated with the placebo control version of Cogmed, improved significantly on various variables. It is probable that active personalized performance coaching in the active Cogmed group during the training period could have made the difference in performance between the 2 groups.

A different explanation for the progression in both groups in our recent study may be that this control version of Cogmed WMT is already challenging enough for children with intellectual disabilities. The WM load in this version has a maximum of 3 items which may place a high demand on the WM capacity of children with MBID. This group may, on average, have a baseline WM capacity below 3, which makes the control version questionable for the MBID-group. This low baseline may also increase the daily duration of the training and consequently decrease the participating children’s motivation. Training is, in general, more difficult for this population. A recent pilot study indicated that protocols containing more training days with shorter training durations per day, may lead to similar or even better training effects compared to the standard protocol, even with less total training time [11]. This is consistent with the general treatment approach for children with MBID. These children may have a shorter attention span and need longer treatment durations before effects – which last for a longer period of time are noticeable [12]. Therefore, based on clinical experience and previous research, a less intensive (i.e. 4 instead of 5 days per week and 5 instead of 8 exercises per day) and more prolonged (i.e. 8 instead of 5 weeks in a row) WMT would probably better fit children with MBID and neuropsychiatric disorders.

In conclusion, robust evidence for transfer effects and treatment benefits of WMT over placebo training are lacking. Double-blind research designs in randomized controlled trials, in which children receive non-specific coaching not based on their actual training performance, might play a crucial role in these findings. Active coaching based on individual training results (comparable to clinical practice) might enhance the efficacy of Cogmed WMT. Furthermore, based on clinical experience and the general treatment approach to children with MBID and neuropsychiatric disorders, it is our opinion that the intensity and duration of WMT is often too stressful. Therefore, this study will examine the efficacy of a less intensive and more prolonged Cogmed WMT in these vulnerable children. Secondly, the potential benefits of active, personalized coaching and feedback during the Cogmed WMT in terms of reducing behavioral symptoms and improving neurocognitive functioning and academic achievements in children with MBID and neuropsychiatric disorders will also be studied.

### Aims

The overall purpose of the study is to gain insight into Cogmed WMT, and in particular to identify which elements may help to develop individually tailored effective treatment possibilities for groups of vulnerable children.

1. The primary aim of this study is to investigate the efficacy of a less intensive but prolonged Cogmed RM WMT with active personalized coaching and feedback in children with MBID and neuropsychiatric disorders, compared to a less intensive but prolonged RM Cogmed WMT without personalized (i.e. only general non-personalized) coaching and feedback, on neurocognitive functioning as measured with the backward block recall task.
2. Secondly, the efficacy of active personalized coaching will be investigated on various other neurocognitive functioning domains (WM and executive functioning), academic achievements (arithmetic and reading), behavioral problems and parenting style.

It is hypothesized that participants who receive a less intensive but prolonged RM Cogmed WMT with active personalized coaching and feedback demonstrate greater improvement on neurocognitive functioning, academic achievements, behavioral problems and parenting style, directly after training and 6-months post baseline, compared to those who receive a less intensive but prolonged RM Cogmed WMT with only general non-personalized coaching and feedback.

## Methods

### Approval

The ethics approval was obtained from the Medical Ethical Committee (NL52647.091.15/METC2015–1618) at Radboud Academic Medical Centre in Nijmegen, the Netherlands.

### Design

Two groups of 26 children will train 8 weeks, 4 days a week for an average of 30 min (5 exercises) each day in a double-blind randomized controlled trial. Parents, children and teachers will be blinded for the group allocation, i.e. 1. personalized coaching and feedback or 2. non-personalized feedback and general coaching. All participants will undergo a behavioral and neurocognitive assessment including academic achievement measures (pre- and post- assessment). The post-assessment will be carried out in the week after the last session, and an evaluation of the training will take place. In this evaluation parents are questioned about their positive and negative experiences with the training. Furthermore they are asked if they would recommend the training to others and to grade their contentment with the training between 0 (very poor) and 10 (very good). Six months after the last training session there will be a follow-up. Selection of participants, screening for eligibility and assessments will be performed at Karakter, Centre for child and adolescent psychiatry. The training will be done at home and/or at school.

- One group will be treated with a less intensive but more prolonged version of the Cogmed WMT, version R/M. They will receive personalized coaching and feedback (5 sessions) based on their actual performance during the training;
- One group will be treated with a less intensive but more prolonged version of the Cogmed WMT, version R/M. They will receive the same amount of coaching time, but without personalized feedback (only non-personalized coaching).

### Setting

Two different types of schools (primary- and secondary special education) in the area of Arnhem/Nijmegen, the Netherlands will be contacted for participation in this study.

### Recruitment

Recruitment will take place among referrals and patients of Karakter child and adolescent psychiatry in the Netherlands. Parents and children will receive detailed written information about the study. If they have any questions about the study, they can call one of the investigators. In addition, participants will be recruited through the Dutch national association for parents of children with developmental disorders (i.e. Stichting Balans). Participants will be recruited through advertisements on the association’s website and magazines. Lastly, participants will be recruited among children visiting special education classes. Primary education school boards will be contacted for participation. If permitted by school board an advertisement will be placed on the school’s website and in the school magazine. The advertisement will be clear about the study objective and design as well as the benefits and the risks of participating in the study. The investigators will only contact parents after they have given permission to their therapist or school board to be informed about the research. Training will be started after signed informed consent has been obtained from both parents and the child (age ≥ 12 years). When informed consent is received by the research team, the investigator will inform the general practitioner and the involved therapist/psychiatrist about the participation.

### Inclusion and exclusion criteria

#### Inclusion

Children participating in this study will be aged between 10 years 0 months and 13 years 11 months with an IQ score between 60 and 85 (mild to borderline intelligence level). They will have been classified with a neuropsychiatric disorder, i.e. ADHD and/or ASD in line with DSM-IV/DSM-5 [13, 14]. Children with comorbid Oppositional Defiant Disorder (ODD) will also be included. Access to a PC with Windows Vista or Windows XP with an internet connection and speakers at home or at school will be required for the training. Children on medication will only be included if there is “room for improvement of the ADHD symptoms” and medication dosages remain stable during study participation.

#### Exclusion

Exclusion criteria are (1) treatment at an inpatient or day treatment clinic, (2) regular use of other medication than for ADHD, (3) psychiatric diagnoses other than ADHD, ASD or ODD, (4) neurological disorders (e.g. epilepsy) in the last 2 years, (5) current or a history of cardiovascular disease, (6) severe motor and/or visual impairment, (7), current participation in another clinical trial, (8) insufficient motivation or time to pursue training (child, parent(s), or aid(es) are too busy or not motivated to participate), (9) medical illness requiring medical treatment.

#### Randomization

Randomization will be used: 1. to avoid bias in the assignment of participants to treatment. 2. to increase the likelihood that known and unknown factors (expectations of the therapy, motivation etc.) are evenly balanced across treatment groups and, 3. to enhance the validity of statistical comparisons across treatment groups. Randomization will be done by an independent person not involved in this research project, and will be provided through sealed envelopes.

Four strata will be constructed based on sex and diagnosis, to enable important prognostic characteristics (the stratification factors) to be balanced between the treatment groups. A block randomization schedule with varying block sizes will be performed separately within each stratum to reduce the possibility of selection bias. By using this method, we will have the most optimally balanced allocation of participants possible among treatment groups. In addition to this the use of varying block sizes will increase the randomness of the group allocation.

Professionals involved with the pre-, post and follow-up assessment will not be informed about the coaching method. Professionals involved in the coaching will not be involved in the pre- post and follow-up assessment. Parents, aides and children will be informed about the weekly contact with their coach by telephone, but will not be informed about coaching-content specifics beforehand. Reason for breaking the blind is that information is important for medical management of the subject. Appropriate documentation will be completed, signed and dated by the investigator with the reason for breaking the code. Participants will be informed about the allocated intervention after the post-treatment assessment.

#### Cogmed WMT

The original Cogmed WMT consists of 13 verbal and visual STM and WM tasks, which are implemented using a computer program (Cogmed, Stockholm, Sweden). A child will complete 8 different task on each training session. An example of a verbal WM task is Decoder. In this particular task, some letters will be said aloud, while 3 letters are shown at the same time with the corresponding letter highlighted. The child needs to remember the letters that he/she hears and select the letters by clicking on them without becoming distracted by the other non-corresponding letters shown on the display. An example of a verbal WM task is Rotating data link. In this particular task a number of lamps will be highlighted in a successive order. The child needs to remember the order. Before the child gives an answer, the entire panel will be turned through 90 degrees. When the program says “go”, the child clicks on the circles in the order in which the lamps were highlighted, however they need to remember that the panel has turned 90 degrees. The program is provided on a compact disc and used by the child on a personal computer at home and/or school, supervised by a parent and/or teacher. Responses are made by clicking on displays using the computer mouse. The difficulty level is automatically adjusted, on a trial-by-trial basis, to match the WM span of the child on each task. Children are assigned a unique ID code and task performance is uploaded in a log file. During the training sessions the child receives positive verbal feedback from the computer. In addition to this high scores are displayed after each task; there is also an “energy” counter which can be used on a fun racing game completed after each training day. The racing game is only included as a reward and does not load on WM. Children have a free choice whether to play the game. After each week of training the child receives a small reward (e.g. doing something fun with dad, choosing dinner).

In the original version children train for a period of 5 weeks (25 days and 200 exercises in total), with an estimated time spent per day between 35 and 50 min. The less intensive but prolonged version has a duration of 8 weeks (30 days and 160 exercises in total), with an estimated time spent per day between 25 and 35 min. In both versions a licensed Cogmed coach will provide detailed personalized coaching and feedback on the child’s performance following a strict protocol, by telephone in 5 sessions with the parent, or aide and also with the participating child. In the original version this means a coaching contact ones a week. To keep additional mental health care costs similar with the original Cogmed version (consisting of five weeks of training and weekly feedback). This means a coaching contact every one and a half week in the prolonged version.

*Cogmed WMT has been developed by Cogmed Cognitive Medical Systems AB (Stockholm, Sweden). Pearson is the official publisher for the Netherlands for Cogmed. BeterBrein provides the educational program to become a licensed Cogmed coach.*

### Interventions

#### Condition 1: A less intensive and prolonged version of Cogmed WM training with active coaching and personalized feedback

This coaching condition aiming to increase performance and motivation is suggested by Cogmed to be the best way to deliver the intervention. During 8 weeks each child will train for an average of 30 min (5 exercises) a day, 4 days a week. The training program consists of WM tasks implemented on a computer program. Three persons are directly involved in the treatment program: (1) The child undergoing the training, (2) parents, teachers or mentors (aides) whose role is to motivate the child during the training, who will have contact with the coach and act as an intermediary between the coach and the child, (3) the licensed Cogmed coach trained by BeterBrein, who will provide detailed personalized coaching and feedback on the child’s performance by telephone in 5 sessions with the parent, and aide (if involved) and the child. Detailed personalized coaching and feedback means explaining in what way the performance can be optimized, by analyzing how and when mistakes are made (the Cogmed coach can log in on the child’s account). For example; performances are better when a child is training in the morning, or a child will perform better when he/she starts with the more difficult exercise(s), or when he/she takes more short breaks. The coach gives this feedback to the child, parent(s) and aid(es). Secondly, motivational aspects are an important part of detailed personalized coaching and feedback. The coach explains why the training is helpful for a better daily functioning and asks the persons involved if everybody sticks to the rewarding plan and if changes have to be made. Furthermore, suggestions in how to motivate the child will be discussed when motivational problems occur. The suggestions are personalized and tailor-made for their specific home situation. The coach, a psychologist or psychological assistant at Karakter, will follow a strict protocol, as part of the training program. According to the protocol the coach will start with a standardized training instruction, in which a demonstration of the training will be given and specific goals (e.g. improvement of memory- or attention span, or better results at school) will be determined. Training days and training times will be discussed and agreements about who will act as aid will be made. Furthermore a rewarding system will be agreed on. During the coaching calls the Cogmed coach will discuss standardized topics such as training progress (e.g. time schedule, training results) by logging in to the Cogmed server to review the child’s exact performance for each training day, to optimize training performance. Also motivation is a standard topic to discuss (e.g. rewarding system, goals).

#### Condition 2: A less intensive and prolonged version of Cogmed WM training without active coaching and personalized feedback, only general non-personalized coaching

This version is exactly the same as the training in the active condition, except for the personalized coaching and feedback. The general non-personalized coaching condition will also start with a standardized training instruction with the same topics, accept for the determination of the specific goals and the use of a rewarding system. The coach will spend the same amount of time with the parents or aid(es) at school and/or child on the telephone, but will not log in on the Cogmed server to see the child’s exact performance. Only concrete training logistics (e.g. training time, training minutes, breaks during the training etc.) will be assessed.

### Measures and procedures

The selection phase will be started once written informed consent has been obtained. Parents will be asked demographic and background information such as previous care and medication use. Parents will also be asked to complete the Diagnostic Interview Schedule for Children (DISC-IV), a highly structured diagnostic instrument designed for use by non-clinicians [15] to assess the presence of ADHD and the exclusion of other comorbid psychiatric disorders. The Social Communication Questionnaire (SCQ) will be used to assess the presence of ASD [16]. These results will be checked against the DSM-IV criteria by an experienced psychologist. Where the most recent results are >2 years old intellectual disabilities will then also be checked before participation through 5 subtests of the WISC-III-NL (Information, Vocabulary, Picture Completion, Block Design, Digit Span) [17]. Children who meet the inclusion criteria will be included in the study. The assessment of neurocognitive functioning domains (WM and executive functioning), academic achievements (arithmetic and reading), behavioral problems and parenting style will be administered at 3 consecutive points: at baseline, directly after treatment and 6 months after treatment. The child will undergo a test battery that will measure neurocognitive functioning, consisting of tests capturing visual [18, 19] and verbal WM [19], sustained attention [20], response inhibition [20] and goal-directed behavior [21]. Academic performance will be measured with a speed reading test [22] and a speed math test [23]. Parents will complete a Dutch translation of the Alabama Parenting Questionnaire (APQ), to measure parenting style [24]. Parents and teachers will complete the Vragenlijst voor Inventarisatie van Sociaal gedrag van Kinderen (VISK, Social Behavior Inventory) [25], the ADHD Vragenlijst (AVL, ADHD Questionnaire) [26] and the Behavior Rating Inventory of Executive Function checklist (BRIEF) [27]. Five (telephone) coaching contacts will take place during the training phase. After completing the training children and parents/teachers in both groups will be questioned about their motivation (e.g. how much fun was it to do) and satisfaction of treatment a scale from 0 to 10. All tests and questionnaires have been selected based on clinical experience with the population and are in line with the international literature. Other aspects taken into account will be the duration of administration, the combination of neurocognitive tests required to determine proximal/near (visual and verbal WM) and distal/far (sustained attention, response inhibition and goal-directed behavior) transfer effects and methodological issues such as presence of Dutch norms and test-retest reliability. See Table 1 for an overview of the assessments and outcome measures.

**Table 1:** Measurements

	Measure	Informant	T0	T1	T2	T3
*Selection fase*	*Shortened version of the Wechsler Intelligence Scale for Children-III-NL:* subtest Information, Vocabulary, Picture Completion, Block Design, Digit Span, which gives an estimation of the Total IQ**	child	*			
	Diagnostic Interview Schedule for Children (DISC-IV): structured diagnostic interview, registered via the internet, Measures presence of symptoms and criteria variables as defined by the DSM-IV. Only domains ADHD (inclusion criterion) and major depression, bipolar disorder, psychotic disorder, conduct disorder and general anxiety disorder (exclusion criteria) are administered.	parent	*			
	Social Communication Questionnaire (SCQ): a quick screening questionnaire of 40 items for autism spectrum disorders.	parent	*			
*Primary outcome*	*Backward block recall:* subtest from the Working Memory Test battery for Children, measures visual working memory.	child		*	*	*
*Secondary outcome*	*Spatial span: subtest from* Automated Working Memory Assessment, measures visual working memory	child		*	*	*
	*Backward digit recall:* subtest from the Working Memory Test battery for Children, measures verbal working memory.	child		*	*	*
	*Listening recall:* subtest from the Working Memory Test Battery for Children, measures verbal working memory.	child		*	*	*
	*Sustained Attention Dots:* subtest from the Amsterdam Neuropsychology Test battery, measures sustained attention.	child		*	*	*
	*Go-Nogo:* subtest from the Amsterdam Neuropsychology Test battery, measures the ability to inhibit automatic responses.	child		*	*	*
	*Comprehension of instruction:* subtest from Nepsy-II-nl, measures the ability to receive, process and execute oral instruction of increasing syntactic complexity.	child		*	*	*
	*Speed math (Tempo Test Rekenen):* measures degree of automation of addition, subtraction, multiplication and decision.	child		*	*	*
	*Speed reading (Een minuut test):* measures technical literacy speed.	child		*	*	*
	*Vragenlijst voor Inventarisatie van Sociaal gedrag van Kinderen (VISK):* measures symptoms of ASD on a 4-point Likert scale.	parent/ teacher		*	*	*
	*ADHD Vragenlijst (AVL):* assess symptoms of ADHD on a 4-point Likert scale	parent/ teacher		*	*	*
	*Behavior Rating Inventory of Executive Function checklist (BRIEF):* 70 item questionnaire designed to assess executive functioning on a 3-point Likert scale in home and school environments.	parent/ teacher		*	*	*
	*Alabama Parenting Questionnaire (APQ):* assess parenting style and indirectly coaching style on a 5-point Likert scale.	parent		*	*	*

*T0 = selection fase, T1 = baseline, T2 = directly after treatment, T3 = follow-up (6 months)*

^**^Intelligence Quotient is only administered if IQ data are not available or when IQ test had been administered more than 2 years ago

Participants can leave the study at any time for any reason if they wish to do so without any consequences. The principal investigator can also withdraw a subject if: The investigator believes that for safety reasons (e.g. in the presence of adverse events) it is in the best interest of the subject to stop treatment, the subject is unwilling to cooperate for reasons not related to the trial treatment, an intercurrent illness emerges of which the severity, duration, or required treatment violated the conditions of the trial. Reasons for withdrawal are recorded. Participants withdrawn from the study for a medical reason will be followed until the adverse events have been resolved.

### Sample size

Data from Fuchs and Fuchs [28] and Wickes [29] were used for the population estimates. In this first meta-analysis the effects of examiner familiarity on children’s test performance was examined. The data for the meta-analysis came from 22 controlled studies involving 1489 subjects. In the typical study, the effect of examiner familiarity raised test performance by .28 standard deviations. Differential performance favoring the familiar examiner condition was greater when subjects were of low socioeconomic status (SES), were tested on comparatively difficult tests, and knew the examiner for a relatively long duration. The second study tested the general hypothesis that test results will be modified by those aspects of the testing situation which are sometimes not carefully controlled or are treated as if they were unimportant. Thirty-six male subjects in two experimental groups and one control group were used to study the effects of perfunctory verbal comments and nonverbal actions on test results. The findings of the study suggest that such comments as ‘Good’ or ‘Fine’ and such actions as smiling and nodding by examiners have a decided effect upon test results. These data is used as population estimates when comparing the effect of active coaching and personalized feedback versus general non-personalized coaching in Cogmed RM WMT. With a sample size of 50 participants (25 per group), our trial will have 79% power to find an effectsize of .40 between groups and a power of 93% to find an effectsize of .50, when co-variating for WM performance at baseline level. An unpublished study in a similar patient group (Roording-Ragetlie S, Klip H, Buitelaar J, Slaats-Willemse D. Working memory training in children with neuropsychiatric disorders and borderline intellectual fucntioning; a randomized controlled trial, unpublished raw data) has shown that four (5%) out 76 participants dropped out of the study. Therefore we will include 2 extra participants in this second intervention study. In total, 52 participants (26 per group) will be included.

### Statistical analysis

Statistical analyses of all data will be undertaken using the SPSS statistical program (SPSS 21.0). Descriptive statistics will be reported for each variable of interest for the two groups (active coaching versus general non-personalized coaching). First univariate analyses will be carried out. An independent-samples t-test will be run to determine if there are differences in age, IQ or severity of ADHD or ASD symptoms (measured with the VISK or AVL questionnaire) between the two groups. A chi-square test will be used to examine whether there is an association between gender, use of medication, whether the intervention occurred in a subject’s home or school and the group (active coaching versus general non-personalized coaching).

The mean, standard error (SE) and 95% confidence intervals will be computed for each parameter. For the primary analyses, an analysis-of-variance (ANOVA) will be conducted on the backward block recall task with group (2 levels) as the between-subject factor and time (pre-, post and follow-up assessment) as the within-subject factor. The group by time interaction will show the treatment effect (*p < 0.05).* For the secondary analyses, a multivariate ANOVA (MANOVA) will be conducted on the other neurocognitive, academic and behavioral scales with again group (2 levels) as the between-subject factor and time (pre, post and follow-up assessment) as the within-subject factor. The group by time interaction will reveal the treatment effect on these variables (*p < 0.05).* In case of withdrawal, discontinuation or missing data (if patients have been missed for any reason (i.e. illness) more than 5 training sessions), intention to treat analyses will be performed.

## Discussion

It is of great importance to undertake a treatment-efficacy study in this large vulnerable population of children with MBID and co-occurring psychiatric disorders. Evidence-based treatment possibilities are scarce for these children, since they are often excluded from participation in scientific research due to the heterogeneity of the population. If a less intensive but more prolonged version of the Cogmed WMT intervention with active coaching and feedback appears to improve neurocognitive functioning, academic achievements and/or behavioral problems, it can be implemented in mental health care and special education schools as an evidence-based intervention for children with MBID and neuropsychiatric disorders. Our study also adds to the literature in that this will be the first study to compare effects of different types of coaching. This aspect is of great value, since several studies have shown significant improvements on both the WM condition as well as the placebo condition if coaching is not personalized. Furthermore, a less intensive and more prolonged WMT would probably better fit these children and might result in an increased training benefit and less dropout. On the other hand one could argue that 4 training sessions a week, for a period of 8 weeks, with a shorter duration of training time a day, is indeed less intensive than 5 training sessions a week, for a period of 5 weeks, with a longer duration of training time a day.

Finally, one strength of this study is the double-blind randomized controlled design, which is the most robust design in trials attempting to establish efficacy. This design not only takes into account near transfer effects, but also far transfer effects. These effects are measured up to 6 months after training.

In this study, the second (non-personalized) coaching condition might be a challenge in clinical practice. Coaches should not be tempted to give any substantive feedback to the parent on how to motivate their child when training difficulties occur. On the other hand, a greater degree of training dropout might take place when non-personalized coaching is performed more rigidly. One limitation of this study is the use of single tasks to assess the generalization effects of WMT. It remains unclear whether any improved performance will be due to an acquired underlying ability and/or reflect practice effects. Therefore the results may have to be interpreted cautiously.

## Abbreviations

ADHD: Attention-deficit/hyperactivity disorder
ANOVA: Analysis-of-variance
APQ: Alabama Parenting Questionnaire
ASD: Autism spectrum disorder
AVL: ADHD Questionnaire; in Dutch: ADHD Vragenlijst
BRIEF: Behavior Rating Inventory of Executive Function checklist
DISC-IV: Diagnostic Interview Schedule for Children
MBID: Mild to borderline intellectual disabilities
MREC/METC: Medical research ethics committee (MREC); in Dutch: medisch ethische toetsing commissie (METC)
ODD: Oppositional Defiant Disorder
RCT: Randomized controlled trial
SCQ: Social Communication Questionnaire
STM: Short-term memory
VISK: Social behavior questionnaire; in Dutch: Vragenlijst voor Inventarisatie van Sociaal gedrag van Kinderen
WM: Working memory
WMT: Working memory training
